# Unexpected pulmonary edema following sitting position craniotomy: A venous air embolism complication

**DOI:** 10.1002/rcr2.70093

**Published:** 2024-12-12

**Authors:** Ahmed Daqour, Abdalkareem Fraij, Dina Khawaja, Tassnim Hussain, Tawfiq Mabhouh, Mohammad Salahaldin, Motaz Saifi, Mohammad Abuawad

**Affiliations:** ^1^ Department of neurosurgery Almakassed Hospital Jerusalem Palestine; ^2^ Department of Medicine Al‐Quds University Jerusalem Palestine; ^3^ Department of Internships Ministry of Health Nablus Palestine; ^4^ Department of Medicine, Faculty of Medicine and Health Sciences An‐Najah National University Nablus Palestine; ^5^ Department of Biomedical Sciences, Faculty of Medicine and Health Sciences An‐Najah National University Nablus Palestine

**Keywords:** craniotomy, pulmonary edema, setting position, venous air embolism

## Abstract

We report a case of a 42‐year‐old female who had non‐cardiogenic pulmonary edema following a setting position craniotomy to remove a left cerebellar pontine angle mass. During the operation, the patient experienced a sudden drop in her end‐tidal CO2 levels, which needed an immediate intervention. After ruling out other potential causes, we determined that air venous embolism was the cause of this unexpected and serious complication. However, the condition was self‐limited and resolved with supportive treatment after approximately 1 week. This case highlights the importance of recognizing and managing the unexpected complications of venous air embolism, as well as how prompt intervention and supportive treatment are critical for improving patient outcomes.

## Graphical abstract

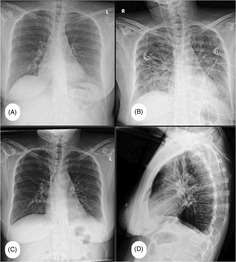

## INTRODUCTION

For many years, certain neurosurgical procedures have been performed in the sitting or semi‐sitting position, especially those involving the posterior fossa or the craniocervical junction.^1^ Challenges in the patient position, such as the increased risk of venous air embolism (VAE), have made the utilization of this position controversial.^2^

VAE is a common complication in certain craniotomy positions. It occurs due to pressure differences between the right atrium and cranial venous sinuses, leading to air inflow when exposed to environmental pressure.^3^ Less serious embolisms can lead to various complications affecting the heart and lungs. One uncommon but significant outcome is the development of noncardiogenic edema following an embolism.^3^

Herein, we present a case of acute pulmonary edema during a retromastoid retrosigmoid craniotomy for an acoustic neuroma due to suspected VAE.

## CASE REPORT

A 42‐year‐old female with no prior medical history presented with left‐sided hearing loss, tinnitus, headaches, vertigo, and brief electric‐like pains along the left trigeminal V1 and V2 nerves distribution for 8 months duration. She has no history of seizures, dental, or orbital issues. Physical exam revealed an absent left corneal reflex, decreased left hearing, positive Romberg sign, and inability to perform tandem gait. Other cranial nerves were intact, with normal extremity movement and reflexes. The brain MRI showed the presence of a lesion at the left cerebellopontine angle with heterogeneous enhancement. Furthermore, she was experiencing high‐tone left sensorineural hearing loss and underwent further evaluation by ENT, followed by neurosurgery consultation for suspected acoustic neuroma.

The surgical procedure involved a left retromastoid retrosigmoid craniotomy to remove the tumour compressing the brainstem, conducted under neuromonitoring in a sitting position. During surgery, the patient experienced decreased end‐tidal CO_2_, with arterial blood gases (ABG) indicating acidosis (pH: 7.27, PCO_2_: 56.4, HCO_3_: 23.7). Immediate measures included packing the wound with wet sterile gauze pads, lowering the patient's head below heart level and aspirating air from the central venous line in the right atrium. Continuous observation ensured adequate ventilation, blood pressure, and heart rate maintenance. Before the extubation, another ABG revealed improvement (pH: 7.35, PCO_2_: 44.7, HCO_3_: 25). Accordingly, the patient was extubated smoothly in the operation room and transferred to the neurosurgical ICU for close monitoring.

**FIGURE 1:**
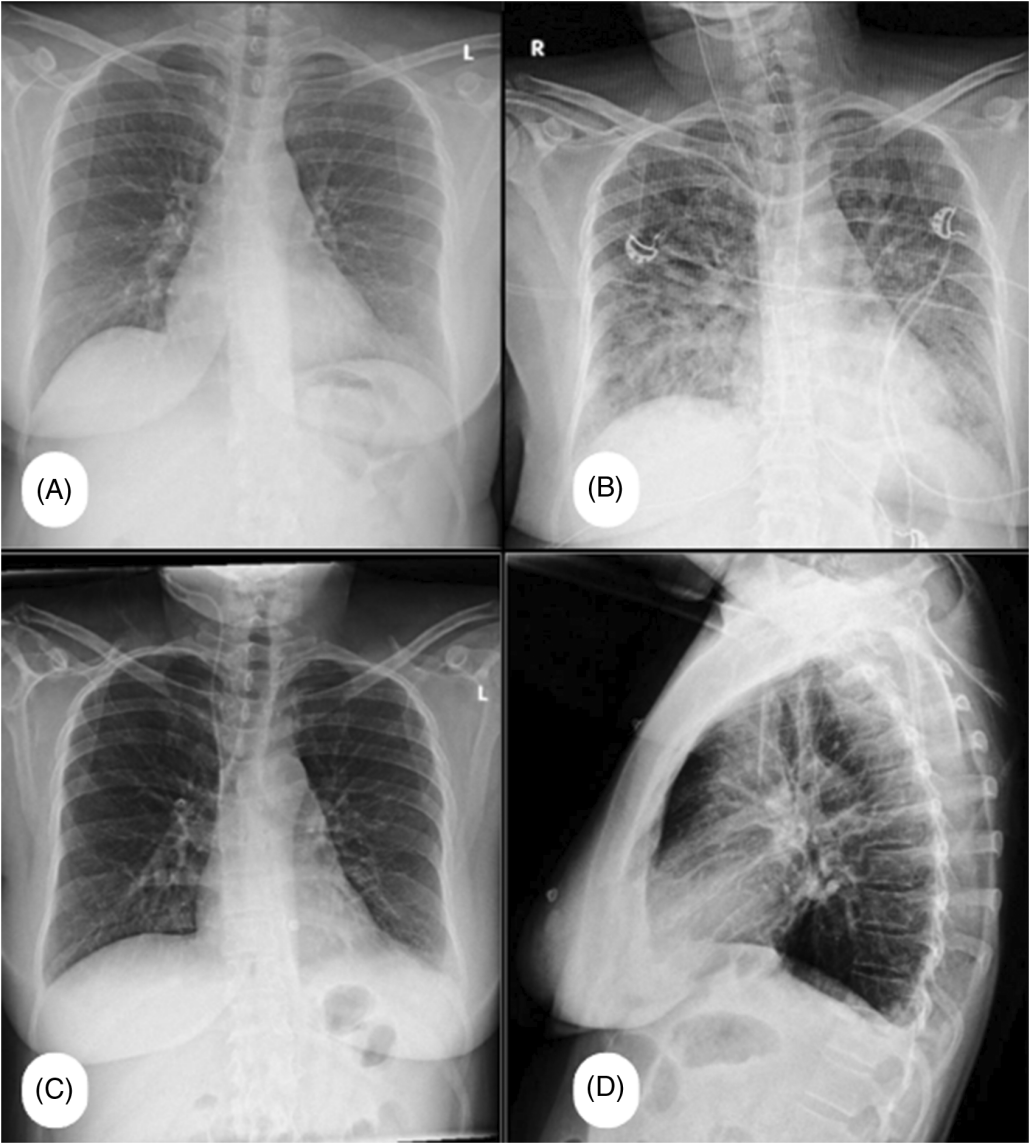
(A) Preoperative normal AP chest x‐ray. (B) Postoperative AP chest x‐ray showing bilateral lung congestion. (C) Seven days post‐operative AP chest x‐rays showing normal appearance of the lungs. (D) Lateral view. AP, antero‐posterior.

**FIGURE 2:**
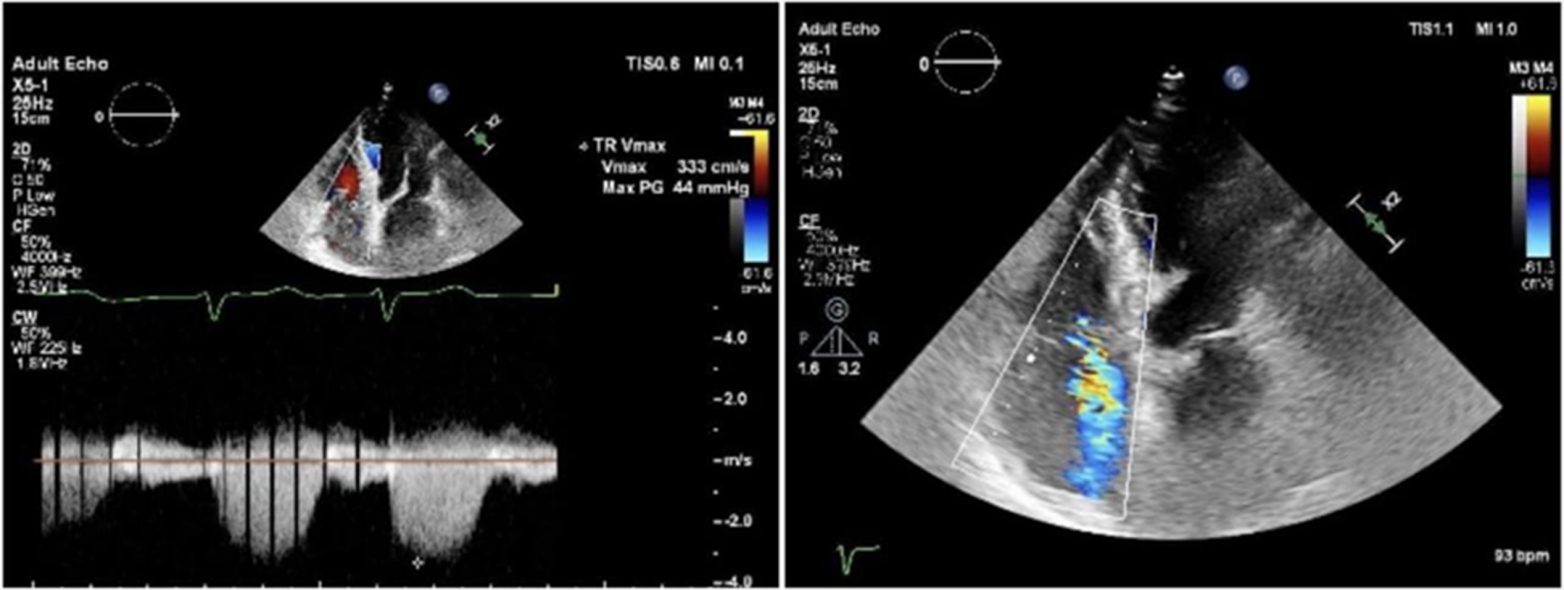
Postoperative echocardiogram showing preserved LV systolic, but dilated RV with reduced contraction and moderate tricuspid regurgitation.

**FIGURE 3:**
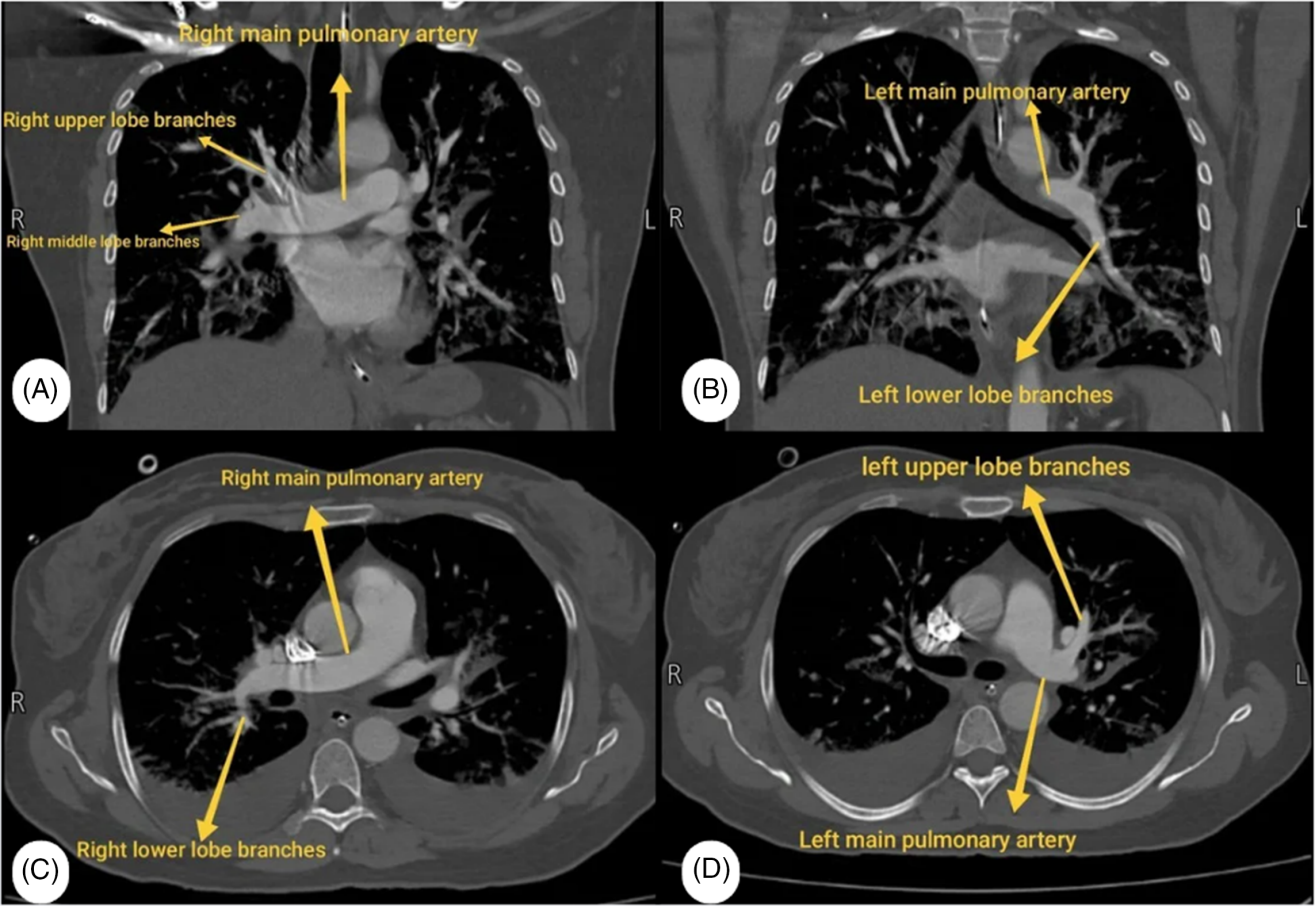
(A, B) Coronal chest CT (mediastinal window) showing the pulmonary arteries. (C, D) Axial view.

Post‐operatively, the patient was conscious and oriented with stable vital signs, except for decreased SpO_2_ (95% on face mask, 88% off O_2_) and tachypnea. Cranial nerve examination showed equal, reactive pupils, bilateral good eye closure, and gag reflex. However, the patient had asymmetrical mouth deviation. Limb movements were normal. Auscultation revealed coarse bilateral crepitations. ABG showed: pH: 7.44, PCO_2_: 27, HCO_3_: 18.2. IV fluids were stopped, and a chest x‐ray showed bilateral lung congestion compared to admission (Figure 1A,B). Moreover, further investigations revealed RSR LAD and flat T waves across all leads (new changes) on ECG, alongside a troponin level of 772 ng/L. A portable echocardiogram showed preserved LV systolic function (EF 55%) but dilated RV with reduced contraction and moderate tricuspid regurgitation, with an RVSP of 54 mmHg (Figure 2). A CT Angiography was free, and showed no evidence of pulmonary embolism or thrombosis (Figure 3). Close observation was maintained with regular evaluation. Following the administration of IV furosemide (40 mg), auscultation revealed a significant improvement in crepitations, which was further confirmed by improvement in follow‐up chest x‐ray, ABG (PH: 7.40, CO_2_: 38, HCO_3_: 23.5), and troponin (becoming 600 ng/L).

The following day, a brain CT scan revealed typical postoperative alterations with successful total tumour resection. In addition, a chest CT scan exhibited consolidation without signs of pulmonary embolism. The Pulmonology team's assessment concluded that there was non‐cardiogenic pulmonary edema.

During examination, the patient was fully conscious, oriented, and alert with a Glasgow Coma Scale (GCS) score of 15/15. Vital signs were stable, and pupils were equal, round, and reactive to light and accommodation. However, left‐sided facial nerve palsy (House‐Brackmann grade 3) and an ataxic gait were observed, necessitating ambulation assistance. Prophylactic enoxaparin was administered. The patient gradually tolerated increased water intake. A subsequent chest x‐ray showed significant improvement. The patient was discharged after 7 days, exhibiting good physical condition, stable vital signs on room air, and clear chest x‐ray (Figure 1C,D). It's worth noting that the pathology report identified the specimen as vestibular schwannoma.

## DISCUSSION

Pulmonary edema can be due to cardiogenic and non‐cardiogenic causes. In this case, cardiogenic cause appeared to be unlikely, as the patient had no prior indications of cardiac disease and no associated hemodynamic symptoms or signs of heart failure, in addition to the pulmonary team's impression. Nevertheless, the potential non‐cardiogenic causes of this case can be separated into: neurogenic pulmonary edema, which was unlikely because it typically occurs due to increased intracranial pressure (ICP), and in this patient, the ICP was within the normal range; brain stem traction, which was absent in our case; plus the normal neurological exam after the surgery and an unremarkable CT scan; or pulmonary embolism, which was rolled out by a normal chest CT scan. Therefore, after excluding all previous causes and looking at the resulting decrease in end‐tidal volume of CO_2_ and changes in ABG results, the diagnosis was consistent with VAE.

Venous air embolism (VAE) is a well‐known complication of neurosurgical procedures performed in the sitting position, with an incidence ranging from 9.3% to 43% and a mortality of 21%.^4^ VAE may manifest intraoperatively with a range of symptoms, including brief and abrupt drops in SpO_2_ and/or EtCO_2_, as well as more severe cardiovascular complications like shock, hypotension, tachycardia, arrhythmias, right heart failure, and even cardiac arrest.^4^ Several mechanisms can cause VAE to affect the small capillaries of the lung, leading to a leaky capillary state and disruptions in the alveolar barrier. This disturbance causes fluid to move into the lung tissue. According to the literature, a few cases of VAE‐induced pulmonary edema have been reported earlier in patients undergoing sitting‐position craniotomy.^5^

The self‐limiting nature of the disease in this case suggests that prompt intervention and supportive treatment were effective. Supportive measures such as oxygen therapy, diuretics, and mechanical ventilation may be necessary in severe cases. Saigal et al. emphasized the significance of prompt recognition and treatment of VAE to minimize morbidity and mortality.^5^ The resolution of symptoms within approximately 1 week indicates a favourable outcome.

In conclusion, this case emphasizes the significance of early detection and management of VAE's complications in neurological surgeries, as well as the critical role that prompt intervention and supportive treatment play in improving patient outcomes.

## AUTHOR CONTRIBUTIONS

As listed above, all authors participated in the manuscript writing in order of their contribution.

## CONFLICT OF INTEREST STATEMENT

None declared.

## ETHICS STATEMENT

The authors declare that appropriate written informed consent was obtained for the publication of this manuscript and accompanying images.

## Data Availability

The data that support the findings of this study are available from the corresponding author upon reasonable request.

## References

[1] Porter JM , Pidgeon C , Cunningham AJ . The sitting position in neurosurgery: a critical appraisal. Br J Anaesth. 1999;82(1):117–128. 10.1093/bja/82.1.117 10325848

[2] Kaye AH , Leslie K . The sitting position for neurosurgery: yet another case series confirming safety. World Neurosurg. 2012;77(1):42–43. 10.1016/j.wneu.2010.12.009 22405382

[3] Giraldo M , Lopera LM , Arango M . Venous air embolism in neurosurgery. Colomb J Anesthesiol. 2015;43:40–44. 10.1016/j.rcae.2014.07.002

[4] Mirski MA , Lele AV , Fitzsimmons L , Toung TJK , Warltier DC . Diagnosis and treatment of vascular air embolism. Anesthesiology. 2007;106(1):164–177. 10.1097/00000542-200701000-00026 17197859

[5] Saigal D , Ganjoo P , Tetarway M , Kiro K . Acute pulmonary edema and thrombocytopenia following venous air embolism during sitting position neurosurgery. Asian J Neurosurg. 2017;12(2):214–216. 10.4103/1793-5482.150223 28484534 PMC5409370

